# Comparative studies on tolerance of rice genotypes differing in their tolerance to moderate salt stress

**DOI:** 10.1186/s12870-017-1089-0

**Published:** 2017-08-17

**Authors:** Qian Li, An Yang, Wen-Hao Zhang

**Affiliations:** 1 State Key Laboratory of Vegetation and Environmental Change, Institute of Botany, the Chinese Academy of Sciences, Beijing, 100093 People’s Republic of China; 2 University of Chinese Academy of Sciences, Beijing, 100049 People’s Republic of China; 3 Research Network of Global Change Biology, Beijing Institutes of Life Science, Chinese Academy of Sciences, Beijing, 100093 China

**Keywords:** Dongdao-4, Osmotic regulation, ROS detoxifying mechanism, Jigeng-88, *Oryza sativa* L., Rice, Moderate salt stress

## Abstract

**Background:**

Moderate salt stress, which often occurs in most saline agriculture land, suppresses crop growth and reduces crop yield. Rice, as an important food crop, is sensitive to salt stress and rice genotypes differ in their tolerance to salt stress. Despite extensive studies on salt tolerance of rice, a few studies have specifically investigated the mechanism by which rice plants respond and tolerate to moderate salt stress. Two rice genotypes differing in their tolerance to saline-alkaline stress, Dongdao-4 and Jigeng-88, were used to explore physiological and molecular mechanisms underlying tolerance to moderate salt stress.

**Results:**

Dongdao-4 plants displayed higher biomass, chlorophyll contents, and photosynthetic rates than Jigeng-88 under conditions of salt stress. No differences in K^+^ concentrations, Na^+^ concentrations and Na^+^/K^+^ ratio in shoots between Dongdao-4 and Jigeng-88 plants were detected when challenged by salt stress, suggesting that Na^+^ toxicity may not underpin the greater tolerance of Dongdao-4 to salt stress than that of Jigeng-88. We further demonstrated that Dongdao-4 plants had greater capacity to accumulate soluble sugars and proline (Pro) than Jigeng-88, thus conferring greater tolerance of Dongdao-4 to osmotic stress than Jigeng-88. Moreover, Dongdao-4 suffered from less oxidative stress than Jigeng-88 under salt stress due to higher activities of catalase (CAT) in Dongdao-4 seedlings. Finally, RNA-seq revealed that Dongdao-4 and Jigeng-88 differed in their gene expression in response to salt stress, such that salt stress changed expression of 456 and 740 genes in Dongdao-4 and Jigeng-88, respectively.

**Conclusion:**

Our results revealed that Dongdao-4 plants were capable of tolerating to salt stress by enhanced accumulation of Pro and soluble sugars to tolerate osmotic stress, increasing the activities of CAT to minimize oxidative stress, while Na^+^ toxicity is not involved in the greater tolerance of Dongdao-4 to moderate salt stress.

**Electronic supplementary material:**

The online version of this article (doi:10.1186/s12870-017-1089-0) contains supplementary material, which is available to authorized users.

## Background

Soil salinity is a global environmental challenge, limiting crop production over 800 million hectares worldwide [1]. The majority of the saline land has arisen from natural events and human intervention, such as release of soluble salts of various types during weathering of parental rocks, the deposition of oceanic salts by wind and rain as well as irrigation containing trace amounts of sodium chloride [2]. Rice is an important cereal that provides 50–80% of daily calorie intake for more than 3 billion people. Rice plants are sensitive to salt stress, particularly at the seedling and reproductive stages. However, rice genotypes differ in their sensitivity to salt stress, and some rice genotypes tolerant to salt stress have been reported, including those of genotypes Pokkali [3] and IR63731–1–1-4-3-2 [4]. Elucidating of the molecular and physiological mechanisms by which rice genotypes respond and adapt to salt stress are pivotal for selecting and breeding rice genotypes capable of growth in the saline soils.

Plants suffering from high salt stress often display symptoms of Na^+^ toxicity due to accumulation of Na^+^, which in turn reduces nutrient acquisition, leading to nutritional imbalances, and oxidative damage [5]. Plants have evolved several mechanisms to cope with these problems. Minimizing Na^+^ toxicity by compartmenting toxic Na^+^ into vacuoles and/or restricting Na^+^ uptake by plants are the most common strategy for plants to tolerate salt stress [2]. For example, greater tolerance to Na^+^ confers barley greater tolerance to salt stress than wheat despite similar foliar Na^+^ concentrations in barely to those of wheat [6].

In addition, plants exposed to salt stress can also suffer from osmotic stress. Therefore, plants have to equip with capacity to tolerate osmotic stress under saline conditions. Salt stress limits plant growth by increasing the osmotic potential of the soil and, thus, decreasing water uptake by the roots. Accumulation of compatible osmolytes in the cytosol, lowering osmotic potential to sustain water absorption from saline soil solutions, is an important salinity tolerance mechanism [7, 8]. Many attempts to molecular breeding plants tolerant to drought have been made by introducing genes that encode key enzymes for biosynthesis of compatible solutes [9].

Increases in activities of enzymes that detoxify reactive oxygen species also contribute to plant tolerance to salinity [10]. For example, Mishra et al. (2013) reported that salt tolerant rice seedlings have a better protection against reactive oxygen species (ROS) by increasing the activities of antioxidant enzymes under salt stress [11]. Transgenic plants overexpressing genes encoding antioxidant enzymes are more tolerant to salt stress than their wild-type counterparts [12, 13].

Several Na^+^ transporters responsible for Na^+^ uptake, translocation and compartmentation have been identified in rice plants, including those OsHKTs, OsHAKs, OsNHXs [14–16]. However, the molecular mechanisms underlying Na^+^ transport from soil solution and within plants remain largely elusive. A comprehensive genome-wide analysis of gene expression in response to salt stress may shed some light on molecular mechanisms responsible for tolerance of rice plants to salt stress. RNA-seq, a high-throughput sequencing technology, was widely used to dissect transcriptomic information. These transcriptome-wide studies have provided new insights on genes and regulatory mechanisms involved in abiotic stresses.

Soil is often referred to as saline one when the electrical conductivity (equivalent to the concentration of salts in saturated soil or in a hydroponic solution) is greater than 4 dS m^−1^, which is equivalent to approximately 40 mM NaCl and yields of most crops are suppressed when grown in such saline soils [2]. However, higher levels of concentration of NaCl (150–200 mM) have been frequently used to study the physiological and molecular mechanisms to saline stress in the literature [3, 17, 18]. Therefore, elucidating the mechanisms underlying response and tolerance to moderate salt stress that is similar to natural saline soils will be of practical implications regarding for breeding crops capable of growing in saline soils. In our previous studies, we collected more than 100 rice genotypes and assessed their tolerance to saline-alkaline stress. Among these rice genotypes, Dongdao-4 is an elite rice genotype that is capable of growing in saline-alkaline soils in the northeast of China, while the genotype Jigeng-88 is a relatively saline-alkaline-sensitive genotype. In addition, we have indicated that Dongdao-4 rice genotype is more tolerant to saline-alkaline stress than Jigeng-88 by more efficient acquisition of iron under saline-alkaline conditions [19]. However, whether the two rice genotypes differ in their tolerance to moderate, neutral salts stress is unclear. In the present study, we made a comparative study on the effects of moderate salt stress on the two genotypes differing in their tolerance to saline-alkaline stress. We further investigated the physiological and molecular mechanisms responsible for salt tolerance in rice plants.

## Results

### Dongdao-4 seedlings are more tolerant to moderate salt stress than Jigeng-88 seedlings

To characterize the differences in salt tolerance between Dongdao-4 and Jigeng-88 plants, three-week-old rice seedlings of the two rice genotypes were exposed to solution supplemented with 20 mM NaCl for one day, and 40 mM NaCl for another day, and then exposed to 60 mM NaCl for one week. We monitored the effects of salt stress on plant growth (Additional file 1: Figure S1), photosynthetic rates (Additional file 1: Figure S2b), and Na^+^, K^+^ concentrations (Additional file 1: Figure S3). Our results showed that, similar to previous results of saline-alkaline stress [19], Dongdao-4 was more tolerant to moderate salt stress than Jigeng-88. Chlorophyll content in Dongdao-4 plants was little affected by saline stress, while a significant reduction in chlorophyll content in Jigeng-88 plants was observed by salt stress, leading to a significantly higher chlorophyll content in Dongdao-4 than in Jigeng-88 when exposed to salt stress (Additional file 1: Figure S2a).

### Dongdao-4 plants accumulated more soluble sugars and Pro

**Fig. 1:**
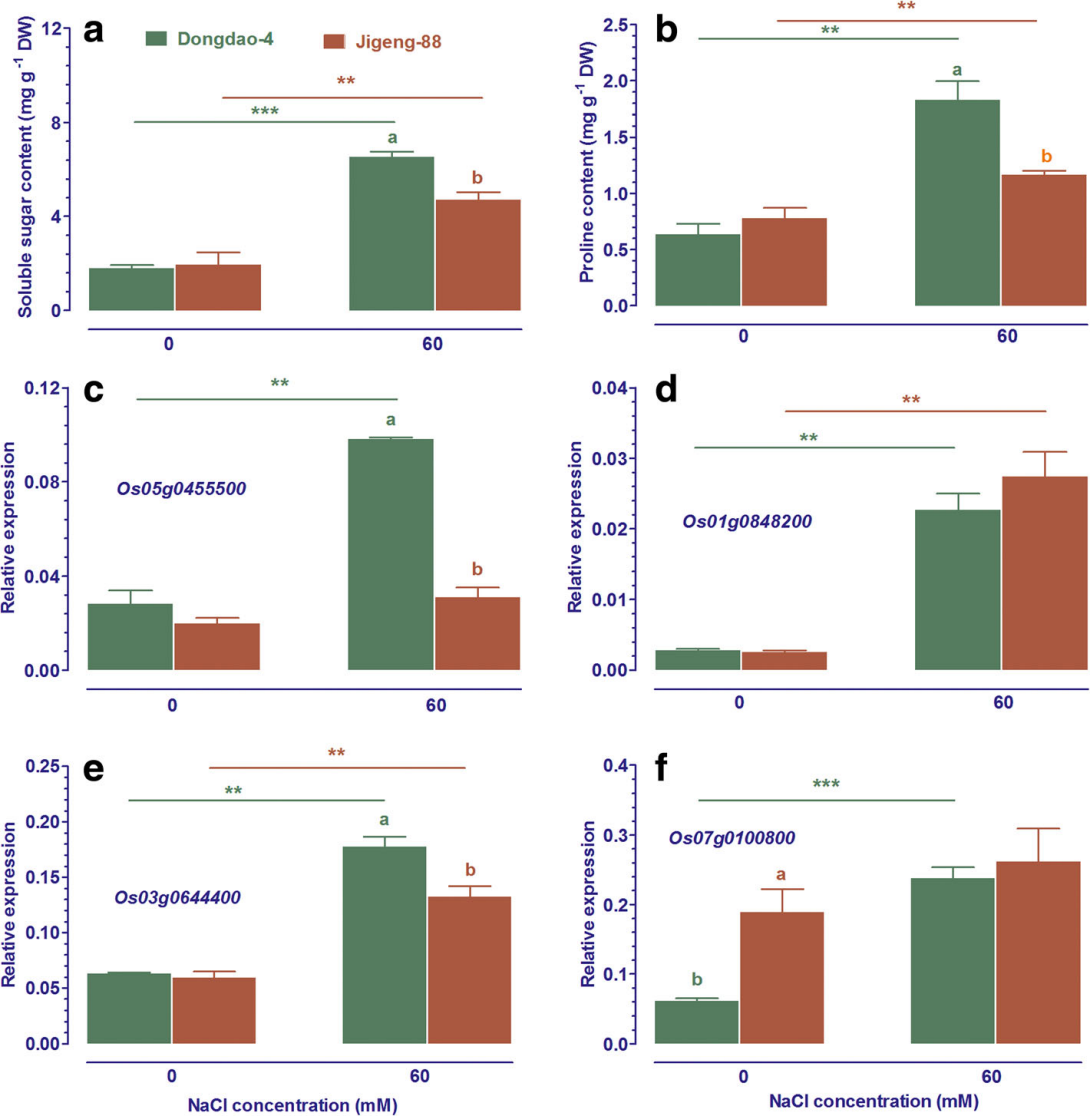
Effect of salt stress on contents of soluble sugar (**a**) and Proline (**b**) in shoot of Dongdao-4 and Jigeng-88 seedlings grown at normal and salt stress conditions. Expression levels of putative proline synthase genes (*Os05g0455500* and *Os01g0848200*; **c** and **d**), transporter genes (*Os03g0644400* and *Os07g0100800*; **e** and **f**) in the two rice genotypes were analysed. Total RNA was extracted from rice seedlings grown under control and salt stress conditions for one week. Transcript levels were measured by real-time PCR. Actin was used as an internal control. *Error bars* are calculated based on three biological replicates. Data are means ±SE (*n* ≥ 4). Means with different letters are significantly different (*P* < 0.05) within the same treatments. *Asterisks* (*) indicate significant differences between control and salt stress of the same genotype which were determined by Student’s t-test (** 0.001 < *P* < 0.01, *** *P* < 0.001)

In addition to toxic effect of Na^+^, plants also suffer from osmotic stress when challenged by salt stress [2]. To cope with osmotic stress, maintenance of turgor pressure by synthesizing, transporting and accumulating low-molecular weight organic compounds is a common strategy [8]. To test whether the enhanced tolerance of Dongdao-4 seedlings to salt stress is related to the capacity to accumulate soluble sugars and Pro, the effect of salt stress on contents of soluble sugars and Pro in Dongdao-4 and Jigeng-88 plants was investigated. The two genotypes exhibited comparable soluble sugars and Pro contents in their shoots in non-salt, control medium (Fig. 1). There were significant increases in shoot soluble sugars and Pro contents of both Dongdao-4 and Jigeng-88 seedlings upon exposure to salt medium. However, the magnitude of salt-induced increases in shoot soluble sugars and Pro contents in Dongdao-4 plants was greater than in Jigeng-88 plants, leading to a significantly higher shoot soluble sugars and Pro contents in Dongdao-4 than that in Jigeng-88 seedlings under salt stress (Fig. 1).

To further elucidate the mechanisms by which Dongdao-4 accumulated greater amounts of Pro than Jigeng-88 under salt stress, the effects of salt stress on the expression of genes responsible for Pro biosynthesis (Δ^1^-pyrroline-5-carboxylate synthase genes, *Os05g0455500* and *Os01g0848200*), Pro transport (*Os03g0644400* and *Os07g0100800*) were investigated. As shown in Fig. 1c, treatment with salt stress led to a great increase in transcripts of *Os05g0455500* in Dongdao-4 seedlings, whereas expression levels of *Os05g0455500* in Jigeng-88 seedlings remained relatively unchanged, leading to a significantly higher expression level of *Os05g0455500* in Dongdao-4 than in Jigeng-88 plants. Exposure to salt stress led to a similar increase in transcripts of *Os01g0848200* in the two genotypes (Fig. 1d). Treatment with salt stress led to a significant increase in transcripts levels of *Os03g0644400* in both genotypes, with the magnitude of increase in Dongdao-4 greater than in Jigeng-88 (Fig. 1e). Expression levels of *Os07g0100800* in Dongdao-4 were significantly lower than those in Jigeng-88 under control conditions (Fig. 1f). Exposure to salt stress led to a significant increase in expression level of *Os07g0100800* in Dongdao-4 plants (Fig. 1f), while expression level of *Os07g0100800* in Jigeng-88 plants was constant upon exposure to salt stress, leading to no differences in expression levels of *Os07g0100800* in the two genotypes under conditions of salt stress (Fig. 1f).

### Effect of mannitol on Dongdao-4 and Jigeng-88

**Fig. 2:**
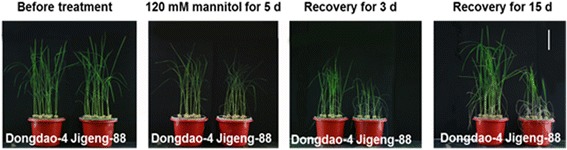
Effects of osmotic stress on growth performance of Dongdao-4 and Jigeng-88 plants after transferred to solution containing40 mM mannitol for 1 day, and 80 mM mannitol for 1 day, and then exposed to 120 mM mannitol for 5 days, finally recovered for another 3 and 15 days. Bars, 10 cm

The greater accumulation of compatible solutes (Pro and soluble sugars) in Dongdao-4 seedlings prompted us to test whether the genotype has greater capacity for osmoregulation, thus conferring it more tolerant to osmotic stress. Therefore, three-week-old seedling of both Dongdao-4 and Jigeng-88 were transferred to solution containing 120 mM mannitol, which approximately equal to the osmolality of solution containing 60 mM NaCl. Upon exposure of the two genotypes to 120 mM mannitol for 5 d, shoot and root biomass and survival rate of both Dongdao-4 and Jigeng-88 were reduced, with the magnitude of reduction in Dongdao-4 plants significantly less than that in Jigeng-88 plants (Fig. 2 and Table 1), suggesting that Dongdao-4 plants are more tolerant to osmotic stress than Jigeng-88.

**Table 1:** Survival rate, Dry shoot biomass, Dry root biomass, Shoot soluble sugar, Shoot Pro of Dongdao-4 and Jigeng-88 plants before, after exposure to medium supplemented with mannitol and recovery from the mannitol treatment. Three-week-old seedlings were transferred to solution containing 40 mM mannitol for 1 day, and 80 mM mannitol for 1 day, and then exposed to 120 mM mannitol for 5 days, finally recovered for 15 days. Data are means ±SE (*n* ≥ 4). Means with different letters are significantly different (*P* < 0.05) within the same treatments. Asterisks (*) indicate significant differences between control and treatments of the same genotype which were determined by Student’s t-test (*0.01 < *P* < 0.05, **0.001 < *P* < 0.01, ***P < 0.001)

	Before treatment		120 mM Mannitol		Recovery	
	Dongdao-4	Jigeng-88	Dongdao-4	Jigeng-88	Dongdao-4	Jigeng-88
Survival rate (%)	100 ± 0.00	100 ± 0.00	100 ± 0.00^***a^	72.92 ± 3.45^b^		
Dry shoot biomass (mg plant^−1^)	113.12 ± 4.47	100.68 ± 3.49	132.18 ± 7.68 ^**a^	83.53 ± 2.99 ^*b^	526.08 ± 18.83 ^***a^	175.03 ± 13.82 ^*b^
Dry root biomass (mg plant^−1^)	12.81 ± 0.12	12.5 ± 0.62	19.95 ± 0.88^**a^	12.73 ± 0.35^b^	66.23 ± 3.85^***a^	16.48 ± 0.66^b^
Shoot soluble sugar (mg g^−1^ DW)	1.79 ± 0.11	1.94 ± 0.43	16.61 ± 0.73^***a^	13.52 ± 0.41^***b^		
Shoot proline (mg g^−1^ DW)	0.67 ± 0.06	0.75 ± 0.06	1.32 ± 0.14^**a^	0.64 ± 0.02^b^		

The two genotypes exhibited a comparable shoot soluble sugars and Pro contents in the absence of mannitol (Table 1). There was a significant increases in shoot soluble sugars and Pro contents in both genotypes upon exposure to the medium supplemented with 120 mM mannitol, whereas the magnitude of treatment-induced increases in shoot soluble sugars and Pro contents in Dongdao-4 plants was greater than that in Jigeng-88 plants, leading to a significantly higher shoot soluble sugars and proline contents in Dongdao-4 than that in Jigeng-88 seedlings under osmotic stress conditions (Table 1).

### Dongdao-4 plants exhibited greater tolerance to oxidative stress

**Fig. 3:**
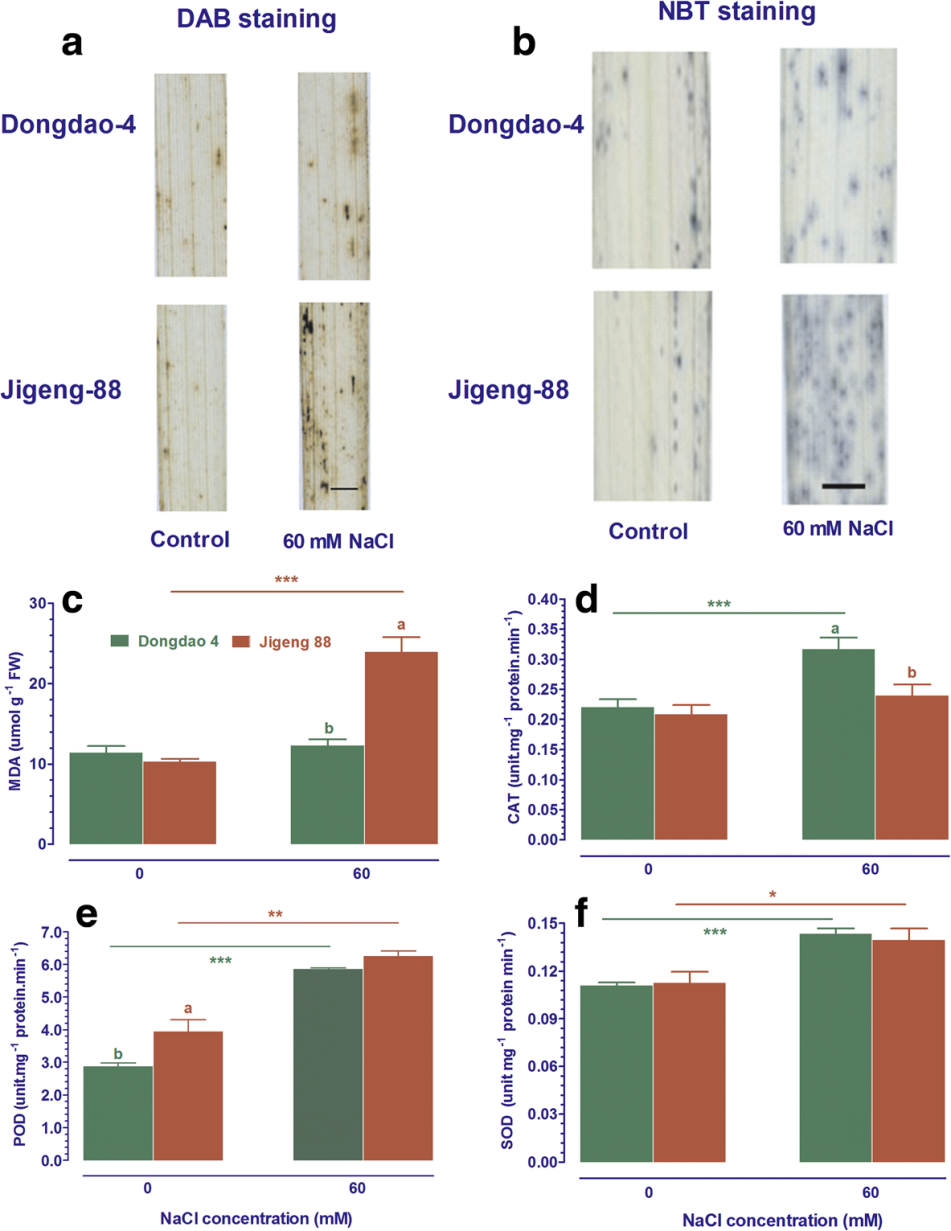
Effect of salt stress on accumulation of reactive oxygen species (ROS) and contents of oxidants and antioxidant enzymes in shoot of Dongdao-4 and Jigeng-88 seedlings. ROS accumulation in Dongdao-4 and Jigeng-88 was detected at 5 day with 3,3′-diaminobenzidine (DAB) (**a**) and nitroblue tetrazolium (NBT) staining (**b**). Bar, 0.1 cm. **c** malondialdehyde (MDA), **d** catalase (CAT), **e** peroxidase (POD), **f** superoxide dismutase (SOD). Data are means ±SE (*n* ≥ 4). Means with different letters are significantly different (*P* < 0.05) within the same treatments. *Asterisks* (*) indicate significant differences between control and salt stress of the same genotype which were determined by Student’s t-test (*0.01 < *P* < 0.05, **0.001 < *P* < 0.01, *** *P* < 0.001)

Plants suffering from abiotic stress often exhibit symptoms of oxidative stress as evidenced by enhanced accumulation of reactive oxygen species (ROS) and malondialdehyde (MDA). Therefore, histochemical analysis was performed to detect ROS accumulation by NBT and DAB staining. As shown in Fig. 3, little ROS accumulation was observed in Dongdao-4 and Jigeng-88 seedlings under control conditions. After treatment with salt stress for one week, an evident ROS accumulation was detected in both Dongdao-4 and Jigeng-88 seedlings, with the levels of ROS in Dongdao-4 lower than Jigeng-88 (Fig. 3a, b). No significant differences in MDA contents in Dongdao-4 and Jigeng-88 seedlings were found when grown in control medium (Fig. 3c). Significant increases in MDA contents in Jigeng-88 seedlings were observed after exposure to salt stress, while Dongdao-4 plants maintain a relatively constant MDA contents when challenged by the identical salt stress, leading to a significantly higher MDA contents in Jigeng-88 than in Dogndao4 plants under salt conditions (Fig. 3c). These results indicate that Dongdao-4 plants are equipped with greater tolerance to the oxidative stress associated with salt stress.

The less accumulation of ROS and MDA in Dongdao-4 seedlings under salt stress prompted us to check the activities of the major antioxidant enzymes. Under control conditions, activities of SOD and CAT were comparable in shoots of Dongdao-4 and Jigeng-88 plants, while activities of POD were higher in shoots of Jigeng-88 than those in Dongdao-4 (Fig. 3d–f). There were marked increases in activities of these enzymes for rice seedlings upon exposure to salt stress, and the treatment-induced increases in activities of POD and SOD had no significant differences in shoots of Dongdao-4 and Jigeng-88 plants (Fig. 3e, f). In contrast, the magnitude of the treatment-induced increases in activities of CAT was greater in Dongdao-4 than in Jigeng-88 plants, such that a significantly higher content of CAT in Dongdao-4 than in Jigeng-88 was observed under salt stress (Fig. 3d). These results suggest that higher activities of CAT may contribute to greater tolerance of Dongdao-4 seedlings to salt stress by counteracting oxidative stress evoked by salt stress.

### RNA-seq analysis

To gain a better understanding of the mechanism underlying salt tolerance of Dongdao-4 seedlings at global transcriptional level, the transcriptome of Dongdao-4 and Jigeng-88 seedlings grown in control and NaCl-supplemented media were investigated by RNA-Seq. In total, 916.86 milloin reads were generated. After trimming adapters and filtering out low quality reads, more than 875.10 million clean reads were retained for futher analysis. Among all the reads, more than 89% had Phred-like quality scores at the Q30 level (an error probability of 0.1%) (Table 2). In addition, principle component analysis (PCA) showed that the two replicates were highly comparable (Additional file 1: Figure S4). These results showed that the quality of throughput and sequencing is high enough for further analysis.

**Table 2:** Summary of sequencing results and their matches in the *Oryza sativa Japonica. cv. Nipponbare* genome

Sample name	Raw reads	Clean reads	Q30%	Total mapped reads
Dongdao-4-control-Shoot-1	55,250,786	54,137,962	92.20	50,926,791
Dongdao-4-control-Shoot-2	58,323,950	57,208,312	92.66	53,987,360
Jigeng-88-control-Shoot-1	58,448,584	56,784,834	89.69	53,001,156
Jigeng-88-control-Shoot-2	54,904,288	53,432,852	90.41	49,824,285
Dongdao-4-salt-Shoot-1	51,848,598	48,385,532	89.52	50,869,413
Dongdao-4-salt-Shoot-2	57,200,640	54,379,656	90.49	44,764,519
Jigeng-88-salt-Shoot-1	62,512,034	57,515,542	90.94	54,147,061
Jigeng-88-salt-Shoot-2	58,009,966	54,798,810	89.09	51,173,210

### Validation of differentially regulated genes (DEGs) by real-time PCR analysis

To validate the data from RNA-sequencing, 15 DEGs were randomly selected for real-time PCR analysis in both genotypes in response to salt stress. The primers of selected genes are listed in Additional file 2: Table S1. A high degree of concordance was observed between the results generated by the two methods (Pearson correlation coefficients R^2^ = 0.6843; Additional file 1: Figure S5).

### Identification of DEGs of Dongdao-4 and Jigeng-88 plants under salt stress

**Fig. 4:**
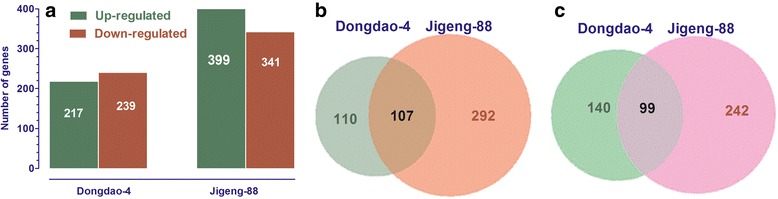
Summary of the numbers of total and shared genes differentially expressed upon treatment by salt stress in shoots of two rice genotypes. **a** The number of genes up- or -down-regulated by salt stress. **b** A venn diagram showing the genes up-regulated by salt stress. The numbers of genes shared and distinct to each genotype are shown. **c** A Venn diagram showing the genes down-regulated by salt stress. The numbers of genes shared and distinct to each genotype are shown

Based on the criteria of a greater than 2-fold change and significance at *P* < 0.05 in t-tests, differentially regulated genes (DEGs) were identified in shoots of Dongdao-4 and Jigeng-88 under salt stress compared with control condidtions. There were more DEGs in salt-sensitive Jigeng-88 than in salt-tolerant Dongdao-4 under conditions of salt stress. More specifically, a total of 456 DEGs were detected in shoots of Dongdao-4 plants, including 217 up-regulated genes and 239 down-regulated genes (Fig. 4a). A total of 740 DEGs were found in shoots of Jigeng-88, including 399 up-regulated genes and 341 down-regulated genes (Fig. 4a). Venn diagram indicates that 110 and 292 genes were specifically up-regulated in shoots of Dongdao-4 and Jigeng-88 seedlings, respectively (Fig. 4b, c). A total of 107 genes were up-regulated in shoots of both Dongdao-4 and Jigeng-88 seedlings, with 3 genes having higher expresssion level in Dongdao-4 than that in Jigeng-88 (Additional file 2: Table S4). Of the down-regulated genes, 140 and 242 genes were specifically inhibited in shoots of Dongdao-4 and Jigeng-88 seedlings, respectively (Additional file 2: Tables S5 and S6). There were 99 genes that commonly suppressed in shoots of both genotypes, with 9 genes having higher expresssion level in Dongdao-4 than that in Jigeng-88 (Additional file 2: Table S7).

### Gene ontology (GO) categorization of DEGs

**Fig. 5:**
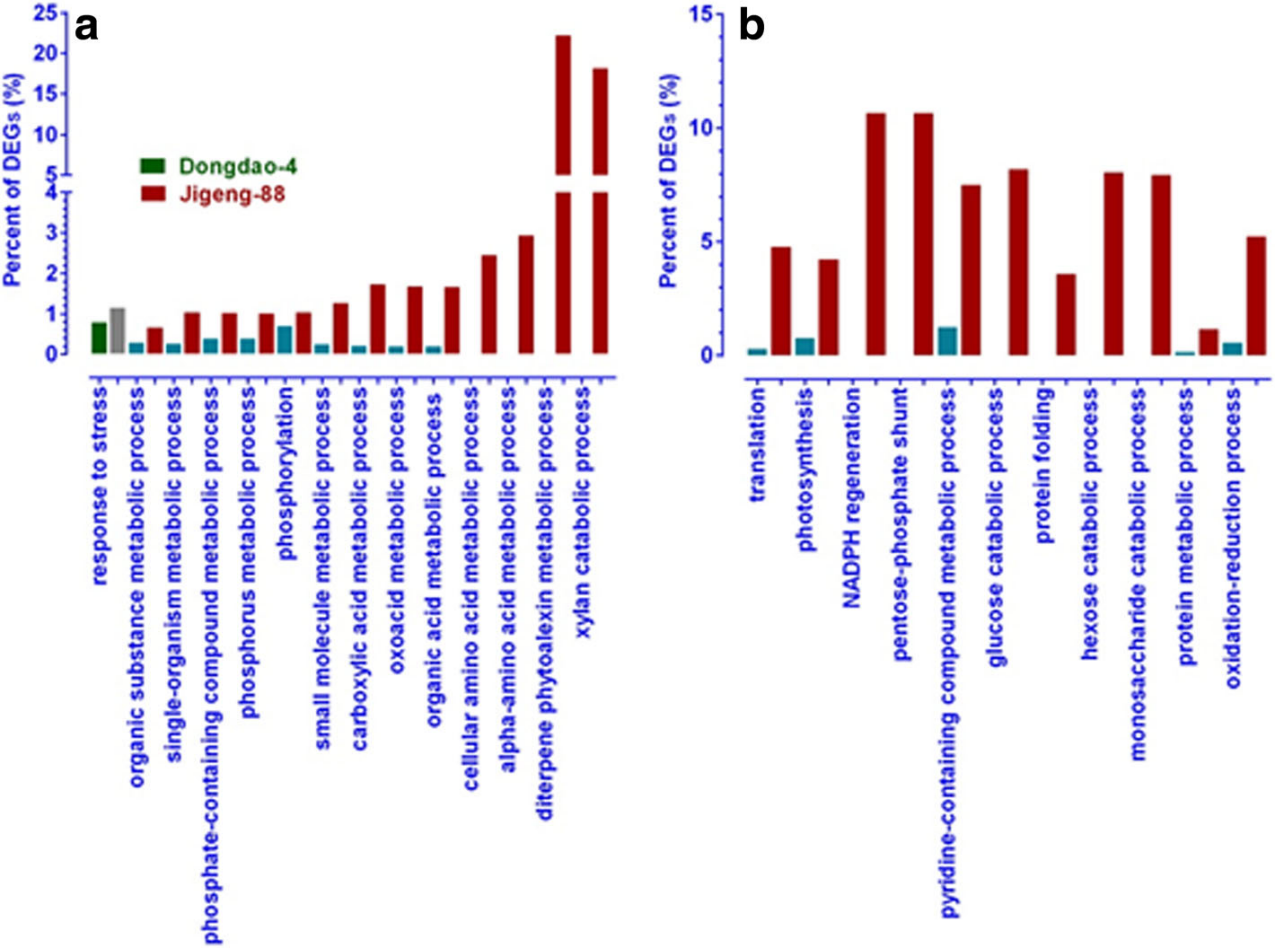
Singnificantly enriched GO terms at biological process ontology level for specifically up- (**a**) and down-regulated (**b**) DEGs in shoots of Dongdao-4 and Jigeng-88. GO terms were defined as significant enriched if false discovery rate (FDR) was ≤0.05. *Green* and *red bars* represent significant enriched GO terms in Dongdao-4 and Jigeng-88, respectively. *Blue* and *grey bars* represent GO terms which were not significantly enriched in Dongdao-4 and Jigeng-88, respectively

To assign functional information to the DEGs that are differently regulated between Dongdao-4 and Jigeng-88 in saline stress, Gene Ontology (GO) analysis was carried out. For the 110 specifically up-regulated in Dongdao-4 seedlings, only one GO term (response to stress) was significantly enriched (Fig. 5a). For the 140 specifically down-regulated genes, no GO term was significantly enriched (Fig. 5b). In Jigeng-88, the 292 specifically up-regulated and 242 specifically down-regulated genes were assigned to 13 and 11 terms, respectively (Fig. 5a and b). The most enriched GO terms of specifically up-regulated genes were diterpene phytoalexin metabolic process, while the most significantly overrepresented terms of specifically down-regulated genes was pentose-phosphate shunt (Fig. 5). For the commonly up-regulated and down-regulated genes, there are only 3 and 9 genes with higher expression level in Dongdao-4 than Jigeng-88, respectively (Additional file 2: Tables S4 and S7). These genes were also used to perform GO analysis and no GO terms were significantly enriched owning to the small percentage overall of the dataset.

## Discussion

Dongdao-4 is an elite rice genotype that is capable of growing in saline-alkaline soils in the northeast of China. Our previous studies revealed that Dongdao-4 plants are equipped with an efficient system for acquisition iron under saline-alkaline conditions, thus conferring tolerance to saline-alkaline stress [19]. In the present study, we evaluated the effect of moderate salt stress on Dongdao-4 plants and compared with that of a saline-alkaline-sensitive genotype, Jigeng-88. Our results demonstrated that, similar to saline-alkaline stress, Dongdao-4 plants were also more tolerant to salt stress than Jigeng-88 plants, as evidenced by less reduction in plant height and shoot biomass (Additional file 1: Figure S1). The greater performance of Dongdao-4 plants may be accounted for by their higher foliar chlorophyll contents and photosynthetic rates than those in Jigeng-88 plants under conditions of salt stress (Additional file 1: Figure S2). Moreover, there were no difference in K^+^ concentrations, Na^+^ concentrations and Na^+^/K^+^ ratio in shoots between Dongdao-4 and Jigeng-88 plants under salt stress (Additional file 1: Figure S3), suggesting that acquisition of Na^+^ and K^+^ may not account for the greater tolerance of Dongdao-4 than Jigeng-88 to salt stress. In addition to Na^+^ toxicity, plants suffering from salt stress also need to deal with osmotic stress. One important finding in the present study is that Dongdao-4 seedlings were capable of accumulating greater amount of soluble sugars, Pro than Jigeng-88 when exposed to moderate salt stress (Figs. 1 and 3). The greater accumulation of soluble sugars and Pro would confer Dongdao-4 plants more effective osmoregulation, thus we further compared transcript profiles of Dongdao-4 with Jigeng-88 in response to salt stress. Our results revealed that Dongdao-4 showed some specific biological processes may underlie the mechanism by which Dongdao-4 has greater tolerance than Jigeng-88. These findings highlight that effective osmoregulation by accumulation of soluble sugars and Pro to maintain water potential and up-regulation of ROS detoxifying systems underlie the greater tolerance of Dongdao-4 to salt stress.

Plants suffering from salt stress have to cope with high concentrations of Na^+^ in the growth medium. Foliar accumulation of Na^+^ in the cytosol may be toxic by inhibiting photosynthesis and other metabolic processes. K^+^ is the most abundant inorganic cation in plants, and plays a diverse role in many physiological processes. Therefore, maintaining an optimal K^+^/Na^+^ ratio is an important indicator of plant tolerance to saline stress. However, there are reports demonstrating independence of salt tolerance and foliar Na^+^ concentration among wheat genotypes [6, 20]. In addition, Pires et al. (2015) observed a high variability of physiological response to salt stress between rice genotypes with similar foliar Na^+^ concentration, suggested that other mechanisms may be responsible for salt tolerance [21]. Almeida et al. (2014) also reported that the maintenance of growth in the presence of 100 mM NaCl can be independent of the exclusion or accumulation of Na^+^ among tomato varieties [22]. In the present study, a comparable Na^+^ concentration and Na^+^/K^+^ ratio was observed in Dongdao-4 and Jigeng-88 when challenged by salt stress (Additional file 1: Figure S3c), suggesting that tolerance of Dongdao-4 plants to salt stress is not achieved by minimizing Na accumulation.

Roy et al. (2014) reported that Na^+^ exclusion may be more effective under conditions of higher salinity, whereas ‘osmotic tolerance’ may be more important in moderately saline conditions [23]. In addition, the introgression of *TmHKT1;5-A* that encodes a Na^+^ transporter from *Triticum monococcum* L. into the durum wheat resulted in a significant improvement in grain yield when growing in saline soils [24, 25]. However, the yield of durum wheat with *TmHKT1;5-A* was similar to that cultivar without the introgressed gene, under low and moderate saline conditions [24, 25], suggesting that osmotic stress has a greater effect on yield of these plants growing in low to moderate saline soils than Na^+^ toxic effect [23]. Therefore, our results highlight, that a higher osmoregulation capacity may confer greater tolerance of Dongdao-4 to moderate, neutral salt stress (i.e., 60 mM NaCl).

Accumulation of compatible solutes (e.g. Pro, raffinose, and glycine betaine) is a common phenomenon for plants to adapt to salt stress by maintaining turgor [2]. In addition, the compatible solutes can also be used to stabilize proteins and cellular structures, and to counteract oxidative stress associated with abiotic stress. In the present study, we found that Dongdao-4 plants accumulated greater amount of soluble sugars and Pro than Jigeng-88 when exposed to salt stress (Fig. 5). We further demonstrated that the expression levels of genes encoding putative Pro synthase and transporters in Dongdao-4 were higher than those in Jigeng-88 under salt stress (Fig. 5), suggesting that the higher expression levels of these genes may underpin the greater accumulation of Pro in Dongdao-4 plants, thus contributing to their greater tolerance to salt stress. The higher Pro and soluble sugar concentration in Dongdao-4 may confer Dongdao-4 plants more effectively osmo regulation, thus accounting for the greater tolerance to osmotic stress than Jigeng-88 (Table 1).

Reactive oxygen species (ROS), as byproducts of photosynthesis, respiration, photorespiration have to maintain in an optimal level to protect plants from oxidative stress and to function as signaling molecules mediating many physiological processes in plants [26, 27]. To avoid excessive accumulation of ROS under salt stress, ROS scavenging mechanisms including enzymatic and non-enzymatic (antioxidants) are often activated in plants. Recent studies reported that an increase in activity of antioxidative enzymes enhanced salt tolerance in rice [11, 28, 29], wheat [30, 31], cotton [32], barley [33], alfalfa [34]. Overexpressing genes encoding antioxidant enzymes enhanced salt tolerance in transgenic plants [12, 13, 35]. In present study, we found that salt stress-induced increases in activities of CAT were greater in Dongdao-4 than in Jigeng-88 plants (Fig. 6), which may allow Dongdao-4 plant to more efficiently regulate ROS, thus conferring it more tolerance to salt stress. No significant differences in the expression level of genes encoding CAT between Dongdao-4 and Jigeng-88 seedlings were found, suggesting that epigenetic and post-transcriptional regulation may underlies the activity of CAT.

To further explore the mechanism underlying the greater tolerance of Dongdao-4 at transcriptome level, we treated Dongdao-4 and Jigeng-88 seedlings with 60 mM NaCl for one week, and analyzed their transcriptome. A total of 110 genes were specifically up-regulated in Dongdao-4 (Additional file 2: Tables S2 and S10). Sun et al. (2013) reported that overexpresion of *GsSRK* in *Arabidopsis* enhanced salt tolerance and higher yields under salt stress [36]. In this study, one gene encoding G-type lectin S-receptor-like serine/threonine-protein kinase (*GsSRK*) was up-regulated (Additional file 2: Table S2). As shown in Additional file 1: Figure S6a, the magnitude of the increase was greater in Jigeng-88 than in Dongdao-4, leading to a signifcantly higher expression levels in Dongdao-4. In addition, nine genes encoding lectin receptor-like kinases that play crucial roles in stress perception [37], were found to be up-regulated. Thaumatin-like proteins (TLPs) is involved in abiotic stresses including salinity and drought [38–42]. Here, one gene encoding thaumatin-like protein was specifically up-regulated by 2.6 fold in Dongdao-4 (Additional file 2: Table S2). Furthermore, WRKY transcription factor 6 and 46 were specifically up-regulated in Dongdao-4 respectively (Additional file 2: Table S2). Validation of *OsWRKY46* by Real-time PCR suggested that the expression level of *OsWRKY46* was up-regulated in both genotypes by salt stress, the increase was much greater in Dongdao-4 than in Jigeng-88 (Additional file 1: Figure S6b). *OsWRKY46* is invovled in regulation of a set of genes associated with cellular osmoprotection and oxidative detoxification under drought and salt stress [43]. To determine if a common transcription factors underlie the specifically up-regulation of genes in Dongdao-4, promoter of these genes were analysed by PlantCARE. As shown in the Additional file 2: Table S10, many motifs have been found in the promoters of these genes by promoter element analysis, such as ABRE (cis-acting element involved in the abscisic acid responsiveness), W-box (WRKY binding site), CGTCA-motif (cis-acting regulatory element involved in the MeJA-responsiveness), MBS (MYB binding site involved in drought-inducibility). However, there are no motif shared by all these genes. Therefore, we speculated that these specifically up-regulated genes may be regulated by multiple transcriptional factors rather than a common transcriptional factor.

A total of 107 genes were up-regulated in both Dongdao-4 and Jigeng-88 (Fig. 4). There are 3 genes with the magnitude of induction being higher in Dongdao-4 than in Jigeng-88, including *Os08g0518900*, *Os12g0564100* and *Os03g0847800*. *Os08g0518900* encoding xylanase inhibitor protein 1 is involved in defence responses that mainly due to biotic stress. Dimkpa et al. (2009) reported that defence response to biotic stress can alleviate abiotic stress conditions, including drought and salinity [44]. These cross-tolerance between abiotic and biotic stress may induce a positive effect in enhancing abiotic tolerance in plants [45–47]. Therefore, the higher transcription levels of genes involved in defence response may contribute to greater tolerance of Dongdao-4 seedlings. MYB transcription factors were reported to be involved in various abiotic stress in plants [48]. *Os12g0564100*, encoding a MYB transcription factor, may also underly the mechanism of greater tolerance to salt stress in Dongdao-4.

Among the commonly suppressed genes in both genotypes, nine genes were inhibited greater in Dongdao-4 than in Jigeng-88 by salt treatment. These genes encoding myo-inositol oxygenase, mannose-1-phosphate guanylyltransferase and protein involving in photosynthesis, are reported to be involved in plant adaption to abiotic stresses. Myo-inositol oxygenase which catalyzes the oxidation of free myo-inositol to D-glucuronate, are related to osmotic balance and possibly to the transport of Na^+^ from root to shoot in the common ice plant *Mesembryanthemum crystallinum* [49]. Cotsaftis et al. (2011) also found that transcript levels of genes encoding myo-inositol oxygenase was significantly down-regulated in salt-tolerant lines FL478 and Pokkli [50]. In addition, Das-Chatterjee et al. (2006) also reported that increased production of myo-inositol through overexpression of the L-*myo*-inositol 1-phosphate synthase gene in transgenic rice provided the plants with increased salinity tolerance [51]. In our results, one gene encoding myo-inositol oxygenase was significantly down-regulated in both genotypes, while the magnitude of reduction in Dongdao-4 was significantly higher than Jigeng-88 (Additional file 1: Figure S6c, Additional file 2: Table S7). This results indicated that decrease of the transcript levels of genes encoding enzymes that degrade or synthesize compatible solutes will be a mechanism to reduce the effects of the imposed stress, thus conferring greater tolerance of Dongdao-4 plants to salt stress.

## Conclusions

In conclusion, we report here that Dongdao-4 was more tolerant to salt stress than Jigeng-88 in spite of an similar Na^+^ concentration in shoot of both genotypes under salt stress. The greater salt-tolerant in Dongdao-4 may be caused by their greater capapcity to synthesize and accumulation of soluble sugars and Pro and higher activities of CAT under salt stress. Our findings demonstrate that the greater tolerance of Dongdao-4 to osmotic stress and efficient ROS detoxifying system underpin the higher tolerance to moderate salt stress than that of Jigene-88. The information about RNA-seq dataset also provides us potent and valuable guidance in underlying the mechanism with which Dongdao-4 has greater salt-tolerance than Jigeng-88.

## Methods

### Plant materials, growth conditions, and stress treatments

Two rice genotypes, *Oryza sativa* L. ssp. Japonica (cv. Dongdao-4 and Jigeng-88), were used in this study. Seeds were germinated in tap water at 37 °C for 2 days and transferred on moist tissue paper for 2 days at 30 °C in the dark. Thereafter the germinated seedlings were transferred to nutrient solution containing (mM): 1.425 NH_4_NO_3_, 0.42 NaH_2_PO_4_, 0.510 K_2_SO_4_, 0.998 CaCl_2_, 1.643 MgSO_4_, 0.168 Na_2_SiO_3_, 0.100 Fe-EDTA, 0.019 H_3_BO_3_, 0.009 MnCl_2_, 0.155 CuSO_4_, 0.152 ZnSO_4_, and 0.075 Na_2_MoO_4_. This hydroponic experiment was carried out in a growth chamber with 30 °C/22 °C (day/night) with 14-h photoperiod, and the relative humidity was controlled at approximately 70%.

For analysis of tolerance to salt stress, Dongdao-4 and Jigeng-88 plants were grown in the culture solution for 3 weeks. Then, half of plants were transferred to culture solution supplemented with 20 mM NaCl for one day, and 40 mM NaCl for one day, and then exposed to 60 mM NaCl for one week. The remaining half of plants was kept in the medium without NaCl as controls. Medium pH was adjusted to 5.8 and the solution was renewed every 3 d for both control and NaCl-supplemented media. To evaluate the effects of osmotic stress on the two rice genotypes, Dongdao-4 and Jigeng-88 seedlings were cultured in the control hydroponic solution for three weeks, and then transferred to the hydroponic solution containing 40 mM mannitol for 1 day, and 80 mM mannitol for 1 day, and then exposed to 120 mM mannitol for 5 days. After treatment, height, shoot biomass, and root biomass were measured. Shoots and roots of the rice seedlings were harvested and oven-dried at 75 °C for 2 days until their weight reached constant for determination of dry weight.

### Determination of Na^+^ and K^+^ concentration

Shoots and roots of rice seedlings were harvested and dried as described in the above section. They were digested with the 6 mL of nitric acid and 2 mL of perhydrol with microwave system (MARS, CEM) after grinding into fine powder. Total ion contents were determined using inductively coupled plasma mass spectrometry (ICAP6300; Thermo Scientific, Waltham, MA).

### Measurements of total root length

To analyze total root length, roots were scanned with an Epson digital scanner (Expression 10000XL, Epson Inc.) and analyzed with the WinRHIZO/WinFOLIA software (Regent Instruments Inc.).

### Measurements of chlorophyll (CHL) concentration

Newly formed leaves were harvested, weighed, and extracted with aqueous ethanol (95% *v*/v) in both Dongdao-4 and Jigeng-88 plants, in order to determine CHL concentration. Absorbance (*A*) readings of the supernatant was recorded at wavelengths of 663 and 645 nm. Total CHL concentration was calculated as 8.02*A*663 + 20.21*A*645, and was expressed as mg chlorophyll g^−1^ fresh weight.

### Measurements of photosynthetic characteristics

Photosynthetic rates of rice seedlings were measured between 8:30–11:30 with a LI-6400 XT portable photosynthesis system equipped with a LED leaf cuvette (Li-Cor, 146 Lincoln, NE, USA). Artificial illumination was applied to the leaves in the chamber from a red-blue 6400-02B LED light source attached to the sensor head with continuous light (1000 μmol m^−2^ s^−1^ photosynthetic photon flux density) and ambient CO_2_ concentration of approximately 500 μmol CO_2_ per mol. At least 15 individual Dongdao-4 and Jigeng-88 plants in each stress treatment were selected for measuring photosynthetic rates.

### Determination of Pro and soluble sugars

Approx. 100 mg dried shoot-materials were extracted in 5 mL 80% ethanol at 80 °C for 1 h, then cooled at room temperature. Approx. 50 mg active carbon were added to the extract for 30 min and then centrifuged at 6000 g for 10 min. The supernatants were filtered and used to determine concentrations of Pro and soluble sugars. Pro contents in rice leaves were determined by the method described previously [48, 52]. In brief, 2 mL filtrate was incubated with 2 mL ninhydrin reagent (2.5% (*w*/*v*) ninhydrin, 60% (*v*/v) glacial acetic acid, 40% 6 M phosphoric acid) and 2 mL of glacial acetic acid at 100 °C for 1 h, and the reaction were terminated in an ice bath. 4 mL toluene was added into the mixtures, followed by vibrating and incubation at room temperature. The absorbance was measured at wavelength of 520 nm using a spectrophotometer (SmartSpecTM Plus, BioRad).

Total soluble sugar content was measured following the methods used previously [48, 52]. Briefly, 5 mL anthrone reagent was added to 1 mL extract incubating at 95 °C for 15 min, and then cooled at room temperature. The absorbance was measured at wavelength of 625 nm using a spectrophotometer (SmartSpecTM Plus, BioRad).

### Visualization of reactive oxygen species (ROS)

The formation of hydrogen peroxide and superoxide anion radicals was detected by 3,3′-diaminobenzidine (DAB) staining and nitroblue tetrazolium (NBT) staining, respectively, as described previously by Wohlgemuth et al. (2002) [53]. In brief, rice leaves from triplicate biological replicates of the samples were first cut into sections (c. 1 cm in length) and then immersed in 40 mL staining solution (0.1% (*w*/*v*) DAB, pH 6.5, or 0.1% (*w*/*v*) NBT, 10 mM sodium azide, 50 mM potassium phosphate, pH 6.4) in a desiccator. Infiltration was carried out by building up a vacuum (∼100–150 mbar) and until the leaves were completely infiltrated. The incubation was conducted in a growth chamber in the dark overnight.

### Determination of malondialdehyde (MDA)

Malondialdehyde (MDA) content in rice leaves was determined following the protocols described by Song et al. (2011) [52]. Briefly, rice leaves were weighed and homogenized in 5 mL of 10% TCA solution, and then centrifuged at 10,000 *g* for 10 min. Thereafter 2 mL supernatant was added in 2 mL of 10% trichloroacetic acid containing 0.6% thiobarbituric acid. The mixture was then incubated in water at 95 °C for 30 min and the reaction was stopped in an ice bath. The absorbance of the solution was measured at 450, 532, and 600 nm, respectively.

### Determination of peroxidase, superoxide dismutase, and catalase activity

Approx. 0.5 g rice leaves were ground thoroughly with a cold mortar and pestle in 50 mM potassium phosphate buffer (pH 7.8) containing 1% polyvinylpyrrolidone. The homogenate was centrifuged at 15,000 *g* for 20 min at 4 °C. The supernatant was crude enzyme extraction. The activities of peroxidase (POD; EC 1.11.1.7), superoxide dismutase (SOD; EC 1.15.1.1), and catalase (CAT; EC 1.11.1.6) were measured using the protocols described by Yang et al. (2012) [48].

### RNA isolation and real-time RT-PCR

Total RNA of shoots and roots was isolated using RNAiso reagent (Takara) and reverse-transcribed into first-strand cDNA with a PrimeScript® RT Reagent kit (Takara). Real-time PCR was performed in an optical 96-well plate with an Applied Biosystems Stepone™ Real-Time PCR system. Each reaction contained 7.5 μL of 2× SYBR Green Master Mix reagent, 0.5 μL of cDNA samples, and 0.6 μL of 10 μM gene-specific primers in a final volume of 15 μL. The thermal cycle used was as follows: 95 °C for 10 min, and 40 cycles of 95 °C for 30 s, 60 °C for 30 s, and 72 °C for 30s. All the primers used for quantitative RT-PCR are listed in Additional file 2: Table S1. The relative expression level was analyzed by the comparative Ct method.

### Total RNA extraction, cDNA library construct ion and Illumina deep sequencing

Total RNA was extracted as described above. The preparation of whole transcriptome libraries and deep sequencing were performed by the Annoroad Gene Technology Corporation (Beijing, PR China). Whole transcriptome libraries were constructed using TruSeq Stranded Total RNA with Ribo-Zero Gold (Illumina, San Diego, CA, USA) according to the manufacturer’s instructions. Libraries were controlled for quality and quantified using the BioAnalyzer 2100 system and qPCR (Kapa Biosystems, Woburn, MA, USA). The resulting libraries were sequenced initially on a HiSeq 2000 instrument that generated paired-end reads of 100 nucleotides.

### Sequencing data analysis

The original image data were transferred into sequence data by base calling, which are defined as raw reads and saved as FastQ files. Prior to data analysis, the raw reads were filtered to obtain the clean reads by removing adaptors, tags of reads and low-quality reads. Then, the remaining high-quality reads were submitted for mapping analysis against the reference genome sequences (ftp://ftp.ensemblgenomes.org/pub /plants/release-30, last accessed November 30, 2015) using Tophat [54]. Differentially expressed genes (DEGs) were identified using the empirical criterion of a greater than 2-fold change and a significant q value (false discovery rate-adjusted *P* value) of <0.05 based on two independent biological replicates. GO enrichment was performed using Cytoscape software (http://www.cytoscape.org, version 2.5.2) with Bingo plugin (http://www.psb.ugent.be/cbd/papers/BiNGO/, version 2.3). Hypergeometric test with Benjamini & Hochberg false discovery rate (FDR) were performed using the default parameters to adjust the *P*-value.

### Statistical analysis

All data were analyzed by the analysis of variance using the SAS statistical software. Significant differences were evaluated using student’s *t*-test.

## Additional files

Additional file 1: **Figure S1.** Effects of salt stress on Dongdao-4 and Jigeng-88 seedlings. (a) Seedling growth performance, (b) Plant height, (c) Dry shoot biomass. Three-week-old rice seedlings grown in normal culture solution were transferred to culture solution supplemented with 20 mM NaCl for one day, and 40 mM NaCl for one day, and then exposed to 60 mM NaCl for one week. Bars, 10 cm. Data are means ±SE (*n* ≥ 4). Means with different letters are significantly different (*P* < 0.05) within the same treatments. Asterisks (*) indicate significant differences between control and salt stress of the same genotype which were determined by Student’s t-test (* 0.01 < *P* < 0.05, ** 0.001 < *P* < 0.01, *** *P* < 0.001). **Figure S2.** Foliar chlorophyll concentration (a) and Photosynthesis rates (b) of Dongdao-4 and Jigeng-88 plants grown at normal and salt stress conditions. Data are means ±SE (*n* ≥ 4). Treatments and statistical analysis were as described in Additional file 1: Figure S1. **Figure S3.** Effects of salt stress on K^+^ concentration (a), Na^+^ concentration (b), Na^+^/K^+^ ratio (c) of shoot in Dongdao-4 and Jigeng-88 seedlings grown at normal and salt stress conditions. Data are means ±SE (*n* ≥ 4). Treatments and statistical analysis were as described in Additional file 1: Figure S1. **Figure S4.** Principal component analysis (PCA) of the RNA sequencing data. The samples (two biological replicates) of each treatment were projected in four principal component; duplicates were projected together, which suggested that the duplicates were more similar. **Figure S5.** Verification of RNA-Seq results by real-time PCR. Correlation between data obtained from RNA-Seq and RT-PCR data. Data are mean ± SE of three replicates. **Figure S6.** Expression levels of *GsSRK* (a), *WRKY46* (b) and *Os06g0561000* (myo-inositol oxygenase) (c) in the two rice plants were analysed. Treatments and statistical analysis were as described in Additional file 1: Figure S1. (DOCX 1123 kb)

Additional file 2: **Table S1.** Primers used in real-time PCR to verify the expression pattern of differentially expressed genes. **Table S2.** Differentially expressed genes up-regulated only in Dongdao-4 under salt stress. **Table S3.** Differentially expressed genes up-regulated only in Jigeng-88 under salt stress. **Table S4.** Differentially expressed genes up-regulated in both Dongdao-4 and Jigeng-88 by salt stress. **Table S5.** Differentially expressed genes down-regulated only in Dongdao-4 under salt stress. **Table S6.** Differentially expressed genes down-regulated only in Jigeng-88 under salt stress. **Table S7.** Differentially expressed genes down-regulated in both Dongdao-4 and Jigeng-88 by salt stress. **Table S8.** List of all the differentially expressed genes in Dongdao-4 by salt stress. **Table S9.** List of all the differentially expressed genes in Jigeng-88 by salt stress. **Table S10.** Promoter analysis of specifically up-regulated genes in Dongdao-4 by PLantCARE (http://bioinformatics.psb.ugent.be/webtools/plantcare/html/). (XLSX 219 kb)
