# Supplementary material for: Comparative studies on tolerance of rice genotypes differing in their tolerance to moderate salt stress
Source: BMC Plant Biol. 2017 Aug 17;17:141. doi: 10.1186/s12870-017-1089-0 (PMC5559854; doi:10.1186/s12870-017-1089-0)
Supplement: Supplementary file 1 — Figure S1. Effects of salt stress on Dongdao-4 and Jigeng-88 seedlings. (a) Seedling growth performance, (b) Plant height, (c) Dry shoot biomass. Three-week-old rice seedlings grown in normal culture solution were transferred to culture solution supplemented with 20 mM NaCl for one day, and 40 mM NaCl for one day, and then exposed to 60 mM NaCl for one week. Bars, 10 cm. Data are means ±SE (n ≥ 4). Means with different letters are significantly different (P < 0.05) within the same treatments. Asterisks (*) indicate significant differences between control and salt stress of the same genotype which were determined by Student’s t-test (* 0.01 < P < 0.05, ** 0.001 < P < 0.01, *** P < 0.001). Figure S2. Foliar chlorophyll concentration (a) and Photosynthesis rates (b) of Dongdao-4 and Jigeng-88 plants grown at normal and salt stress conditions. Data are means ±SE (n ≥ 4). Treatments and statistical analysis were as described in Additional file 1: Figure S1. Figure S3. Effects of salt stress on K+ concentration (a), Na+ concentration (b), Na+/K+ ratio (c) of shoot in Dongdao-4 and Jigeng-88 seedlings grown at normal and salt stress conditions. Data are means ±SE (n ≥ 4). Treatments and statistical analysis were as described in Additional file 1: Figure S1. Figure S4. Principal component analysis (PCA) of the RNA sequencing data. The samples (two biological replicates) of each treatment were projected in four principal component; duplicates were projected together, which suggested that the duplicates were more similar. Figure S5. Verification of RNA-Seq results by real-time PCR. Correlation between data obtained from RNA-Seq and RT-PCR data. Data are mean ± SE of three replicates. Figure S6. Expression levels of GsSRK (a), WRKY46 (b) and Os06g0561000 (myo-inositol oxygenase) (c) in the two rice plants were analysed. Treatments and statistical analysis were as described in Additional file 1: Figure S1. (DOCX 1123 kb) [file 12870_2017_1089_MOESM1_ESM.docx]

**Figure S1.** Effects of salt stress on Dongdao-4 and Jigeng-88 seedlings. (A) Seedling growth performance, (B) Plant height, (C) Dry shoot biomass. Three-week-old rice seedlings grown in normal culture solution were transferred to culture solution supplemented with 20 mM NaCl for one day, and 40 mM NaCl for one day, and then exposed to 60 mM NaCl for one week. Bars, 10 cm. Data are means ±SE (n≥4). Means with different letters are significantly different (*P*<0.05) within the same treatments. Asterisks (*) indicate significant differences between control and salt stress of the same genotype which were determined by Student’s t-test (*** *P*<0.001).

**Figure S2.** Foliar chlorophyll concentration (A) and Photosynthesis rates (B) of Dongdao-4 and Jigeng-88 plants grown at normal and salt stress conditions. Data are means ±SE (n≥4). Means with different letters are significantly different (*P*<0.05) within the same treatments. Asterisks (*) indicate significant differences between control and salt stress of the same genotype which were determined by Student’s t-test (*0.01<*P*<0.05, *** *P*<0.001).

**Figure S3.** Effects of salt stress on K^+^ concentration (A), Na^+^ concentration (B), Na^+^/K^+^ ratio (C) of shoot in Dongdao-4 and Jigeng-88 seedlings grown at normal and salt stress conditions. Data are means ±SE (n≥4). Means with different letters are significantly different (*P*<0.05) within the same treatments. Asterisks (*) indicate significant differences between control and salt stress of the same genotype which were determined by Student’s t-test (**0.001<*P*<0.01, *** *P*<0.001).

**
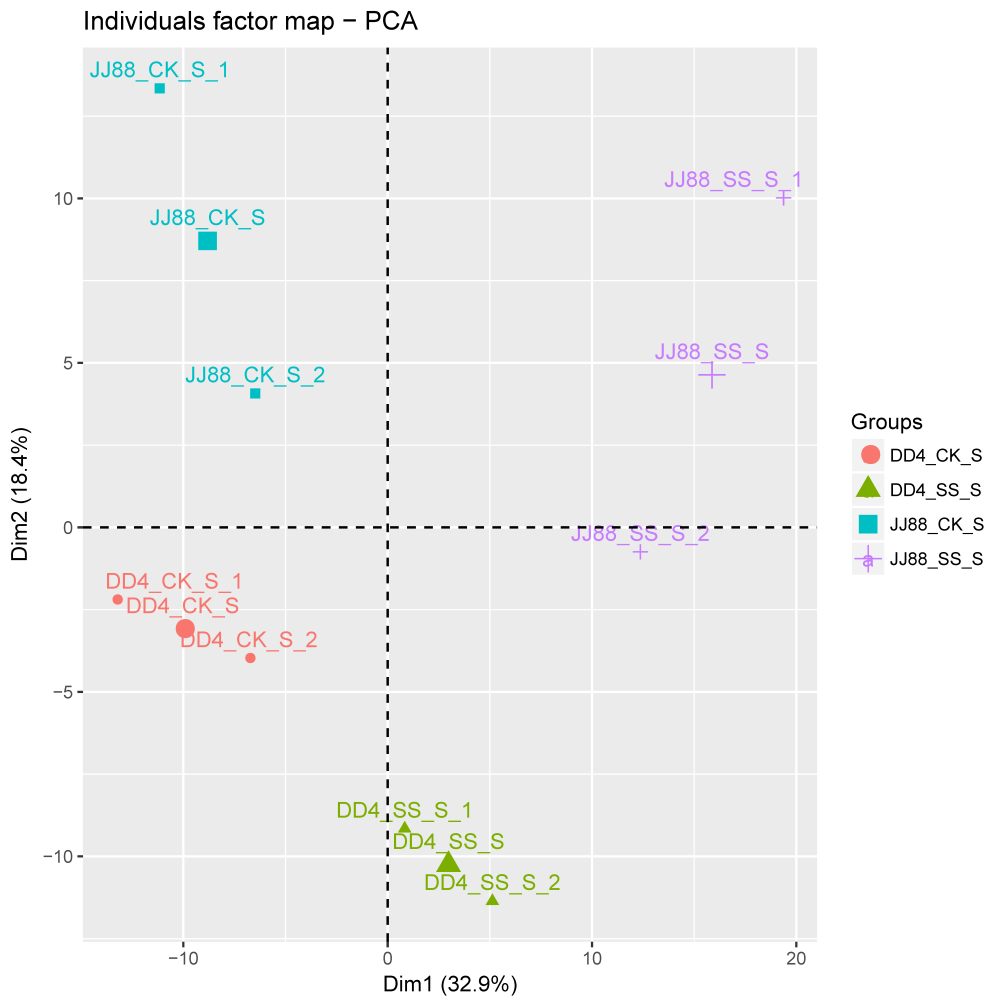
**

**Figure S4.** Principal component analysis (PCA) of the RNA sequencing data. The samples (two biologocal replicates) of each treatment were projected in four principal component; duplicates are projected together, which suggests that the duplicates are more similar.

**Figure S5** Verification of microarray results by real-time PCR. Correlation between data obtained from RNA-Seq and RT-PCR data. Data are mean ±SE of three replicates.

**Figure S6** Expression levels of *OsGsSRK* (A), *OsWRKY46* (B) and *OsMIPS* (C) in the two rice plants were analysed. Total RNA was extracted from rice seedlings grown under control and salt stress conditions for one week. Transcript levels were measured by real-time PCR. Actin was used as an internal control. Error bars are calculated based on three biological replicates. Data are means ±SE (n≥4). Means with different letters are significantly different (*P*<0.05) within the same treatments. Asterisks (*) indicate significant differences between control and salt stress of the same genotype which were determined by Student’s t-test (**0.001<*P*<0.01, *** *P*<0.001).
